# Neuroendocrine neoplasms of the thymus

**DOI:** 10.3389/fimmu.2024.1465775

**Published:** 2024-08-29

**Authors:** Paul D. Barone, Chen Zhang

**Affiliations:** Department of Pathology and Laboratory Medicine, Weill Cornell Medicine, New York, NY, United States

**Keywords:** neuroendocrine neoplasm, thymus, carcinoid, large cell neuroendocrine carcinoma, small cell carcinoma

## Abstract

Neuroendocrine neoplasms of the thymus (tNENs), including typical carcinoid, atypical carcinoid, large cell neuroendocrine carcinoma, and small cell carcinoma, are rare tumors with scarce clinical and pathological data available in the literature. They share many common features with neuroendocrine neoplasms in other organs, such as those in the lungs, while demonstrating some distinct clinical and pathological features. This review aims to give an updated overview of each category of tNENs, focusing primarily on the pathologic diagnosis and differential diagnosis of these tumors.

## Introduction

Neuroendocrine neoplasms of the thymus (tNENs) consist of a spectrum of rare neuroendocrine epithelial tumors, accounting for less than 5% of total primary tumors in the thymus and mediastinum, and about 0.4-0.9% of neuroendocrine neoplasms of all organs (1–3). Due to the rarity of the tumors, clinical and pathological data are scarce.

The current World Health Organization (WHO) classification of tNENs follows its pulmonary counterpart and separates these tumors into typical carcinoid (TC), atypical carcinoid (AC), large cell neuroendocrine carcinoma (LCNEC), and small cell carcinoma (SmCC) (**Table 1**). Recent whole-genome sequencing data on tNENs demonstrated three distinct molecular subgroups with low, intermediate, and high copy number instability (CNI) scores, respectively. The majority of TCs with excellent clinical prognosis fell into the CNI-low subgroup and all SCCs with worse prognosis fell into the CNI-high subgroup. The ACs and LCNECs were found variably located in all three subgroups, which corresponds to the variable morphologic features and clinical prognosis of these tumors. Based on these findings, a grading system to incorporate morphology with molecular characteristics has been proposed for patient stratification and prognostication (4).

**Table 1:** Summary of thymic neuroendocrine tumors.

	Incidence	Mitosis (/2mm^2^)	Necrosis	Ki-67 index	IHC NE marker expression	CNI score	Treatment	Prognosis (5-year survival)
TC	20%	<2	Absent	Low, <20%	Diffuse	Low	Surgical resection, with/without post-surgical radiotherapy	50-100%
AC	60-70%	2-10	Absent or focal	Elevated up to 30%	Diffuse	Variable, majority low to intermediate	Surgical resection, with/without post-surgical chemoradiotherapy	20-70%
LCNEC	<10%	>10, often >20	Extensive	>30%, generally 40-80%	Diffuse or focal	Variable, majority intermediate to high	Sometimes require pre-surgical induction chemotherapy; surgical resection with post-surgical chemoradiotherapy	0-60%
SmCC	<10%	>10, usually >50	Extensive	>50%, often 80-100%	Diffuse, focal or none	High	Commonly unresectable; chemoradiotherapy	0%

CNI, chromosomal instability; IHC, immunohistochemistry; NE, neuroendocrine; LCNEC, large cell neuroendocrine carcinoma; SmCC, small cell carcinoma; TC, typical carcinoid.

The tNEN are morphologically indistinguishable from pulmonary neuroendocrine neoplasms (pNENs) in each category (5). The differentiation from pNENs relies almost entirely on clinical information and radiological studies. Currently available epidemiology data show that around 25% of carcinoids of both thymus and lung are associated with multiple endocrine neoplasia type 1 (MEN-1) (6), but with male predominance in the thymus and female predominance in the lung (7). AC is the most common NEN in the thymus, while TC and SmCC are the dominant types in the lung. Numerous studies have shown that cigarette smoking plays a critical role in the pathogenesis of SmCC and LCNEC in the lungs, while a close relationship between smoking and tNENs has not been demonstrated.

## Typical carcinoid

TC is also known as low-grade neuroendocrine tumor (NET) or grade 1 NET. About 20% of tNENs are TCs. Half of the patients with TC present with chest pain, cough, dyspnea, and other respiratory symptoms. Around 30% of the patients demonstrate paraneoplastic manifestations such as Cushing syndrome due to adrenocorticotropic hormone (ACTH)-like hormone production (8) and hypercalcemia due to parathyroid hormone-related peptide (PTHrP) production (9). Hyperparathyroidism is commonly seen in patients with MEN-1 (10). Inappropriate production of antidiuretic hormone or atrial natriuretic peptide has also been described in patients with thymic TC (11). Carcinoid syndrome is rarely seen in association with thymic TC (2, 7).

Radiologically and macroscopically, thymic TC is difficult to distinguish from other thymic neoplasms such as thymoma. They are usually unencapsulated, with well-defined borders or with invasive growth patterns. The tumor size ranges from 2 cm to 20 cm. TC with paraneoplastic manifestations such as Cushing syndrome tend to be discovered early with smaller tumor sizes. The cut surface of TC is grey-white and firm, commonly with slight gritty consistency due to calcifications (1). The cut surface of the oncocytic variant is usually tan-brown (1, 7).

**Figure 1:**
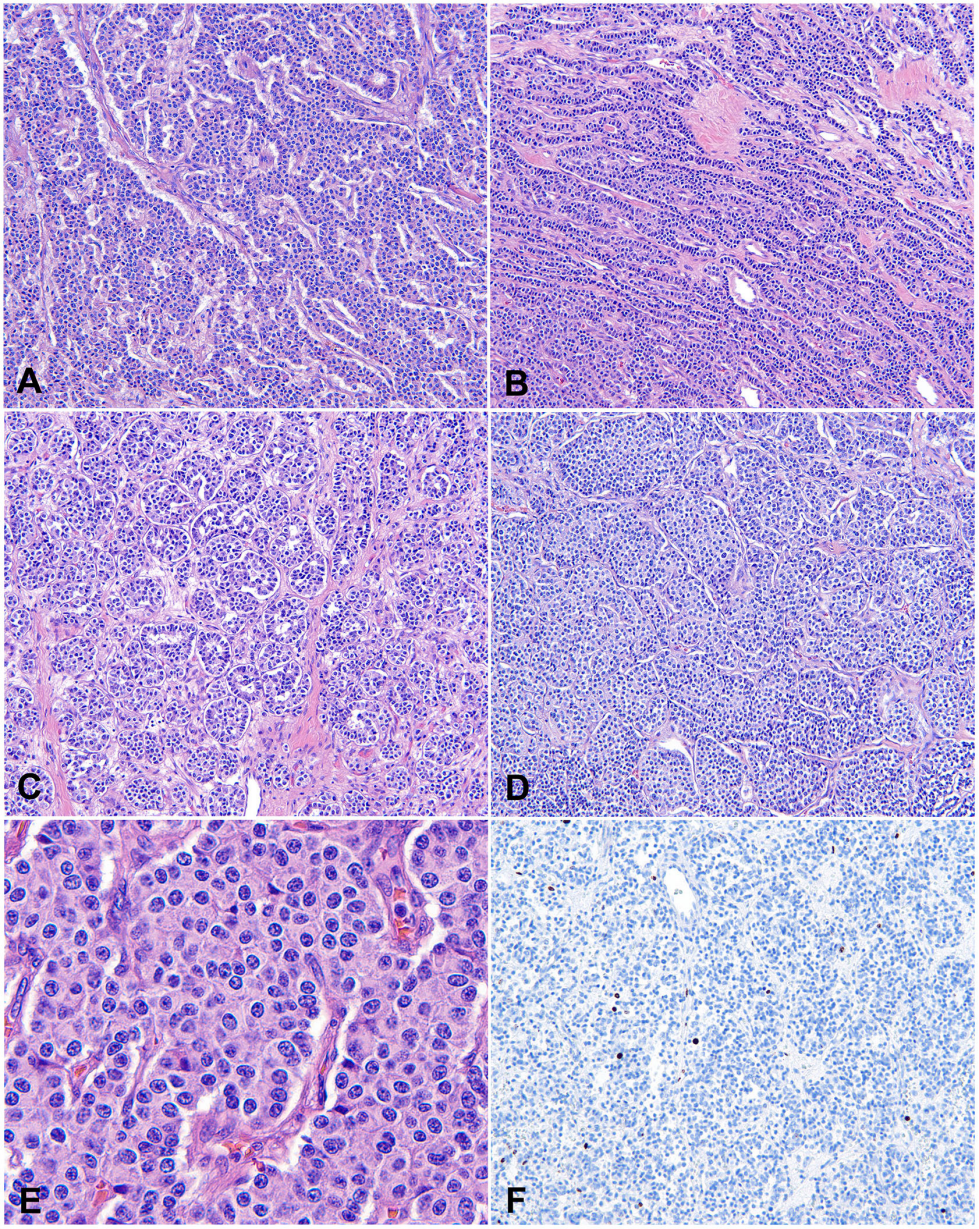
Typical carcinoid (TC). **(A-D)** Low to intermediate magnification photomicrographs of TCs show that tumor cells are arranged in trabeculae **(A)**, cords and ribbons **(B)**, glandular structures **(C)**, or solid nests **(D)**, with fine fibrous septa and rich, delicate vasculatures. Magnification **(A-D)**: 40X. **(E)** High magnification view of TC comprising bland tumor cells with a moderate amount of pale eosinophilic cytoplasm, round to oval nuclei, and finely granular, salt-and-pepper chromatin. The cells are arranged in organoid solid nests separated with delicate vascular stroma. There is no mitosis or necrosis. Magnification **(E)**: 400X. H&E stain **(A-E)**. **(F)** The Ki-67 index is low (<2%).

Microscopically, thymic TC consists of tumor cells with neuroendocrine cytologic features, including moderate to abundant pale eosinophilic cytoplasm and finely granular, salt-and-pepper nuclear chromatin. The tumor nuclei are round to oval with minimal pleomorphism. Mitotic activity is less than 2 mitoses/2 mm^2^, which corresponds to 10 high power fields (HPF) in most microscopes but varies depending upon the manufacturer and model of the microscope. No necrosis is present. Tumor cells are arranged in trabeculae, ribbons, cords, rosettes, glandular structures, or solid nests, with fine fibrous septa and rich, delicate vasculatures (**Figures 1A-E**). Rare variants of TC include spindle cell (12), oncocytic (1), mucinous (13), angiomatoid (14), and sarcomatous with myoid, chondroid, or osseous differentiation (15, 16). Lymphovascular invasion is common. Metastasis in regional lymph nodes is common (up to 50% of patients at presentation). Distant metastasis can also happen. Bones and lungs are the most common metastatic locations, followed by liver and other abdominal organs (1, 2).

The immunohistochemical profile of thymic TC is similar to its pulmonary counterpart. All cases of TC are diffusely positive for the common neuroendocrine markers such as synaptophysin, chromogranin A and INSM1. Most (over 80%) are positive for cytokeratins such as AE1/AE3 and Cam 5.2 (1). The Ki-67 index is generally less than 20% (**Figure 1F**), although it is not one of the diagnostic criteria in the current WHO classification of tNENs. In small and crushed biopsy specimens, the Ki-67 staining can help rule out high-grade NENs such as SmCC, which show a high Ki-67 index of over 50%, often 80-100%. Molecular studies show low mutation burdens with a high prevalence of mutations in chromatin remodeling and histone modification-related genes and few chromosomal aberrances in TC (2).

The most encountered differential diagnosis of thymic TC is thymic/mediastinal involvement of pulmonary TC. As mentioned in the introduction, clinical information and radiology studies are usually sufficient to tell the primary location of the tumor. Histological features of thymic TC are identical to those of pulmonary TC. A minority (around 30%) of thymic TC show focal or diffuse reactivities to PAX-8, and they are almost always negative for TTF-1. Pulmonary TC is the opposite, with most (70 -90%) of them reactive to TTF-1 but usually nonreactive to PAX-8 (17, 18). Due to the variable expression levels of TTF-1 in pulmonary TC, a negative TTF-1 test alone cannot be used to favor a thymic origin. Other differential diagnoses can be encountered in some variants of thymic TC. For example, spindle cell TC can be mistaken for type A thymoma; mucinous variant needs to be differentiated from metastatic mucinous adenocarcinoma; sarcomatous variant can mimic true sarcomas such as synovial sarcoma. Most of these differentials can be solved using a brief panel of immunohistochemical stains, for example, pancytokeratin for type A thymoma, CK20 and CDX-2 for metastatic mucinous adenocarcinoma, and TLE-1 or CD99 and SS18-SSX for synovial sarcoma.

The primary treatment choice of thymic TC is surgical resection. Postsurgical radiotherapy has also been shown to improve survival. Chemotherapy alone or postsurgical chemotherapy has not demonstrated consistent benefits on survival rates. The 5-year survival rate of thymic TC ranges from 50-100%, with a median survival of 126 months (7, 19, 20). A recent retrospective analysis of 146 patients with thymic neuroendocrine neoplasms did not identify a significant difference in disease-free survival between thymic TC and thymic AC; however, there was a clear difference between the carcinoid categories and the neuroendocrine carcinomas (21).

## Atypical carcinoid

**Figure 2:**
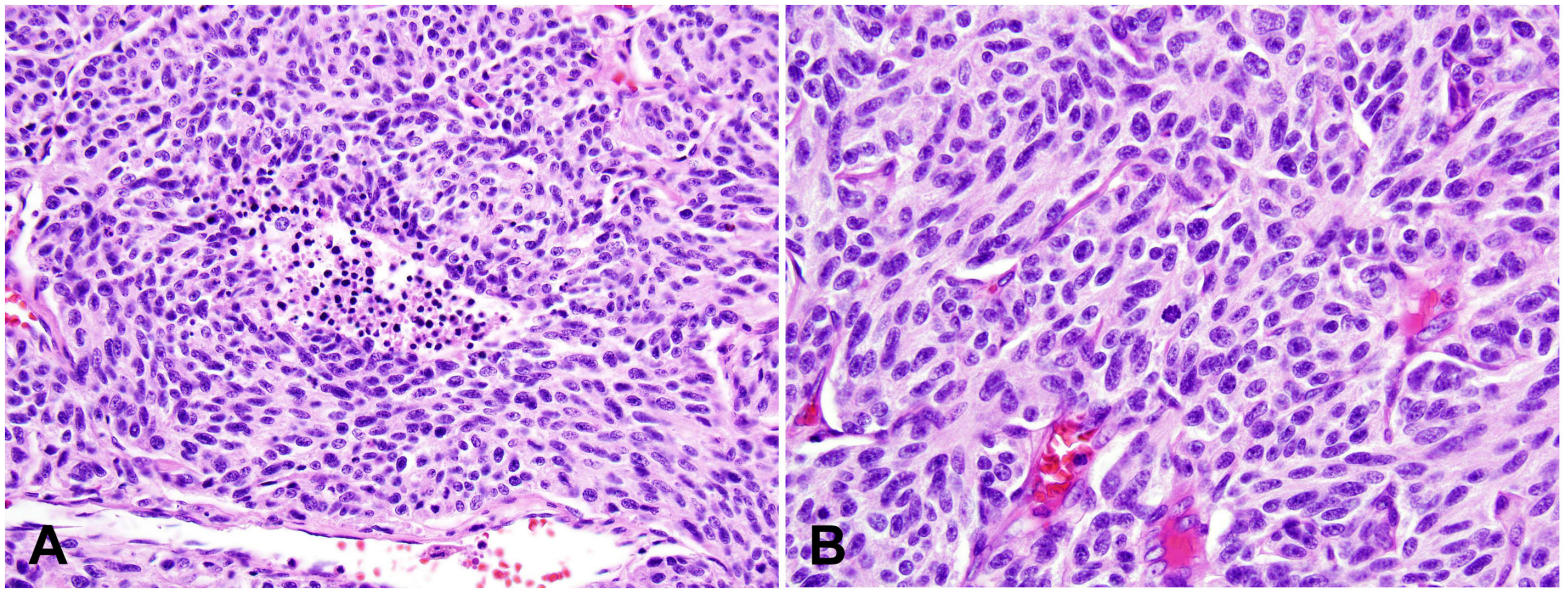
Atypical carcinoid (AC). **(A)** Intermediate magnification photomicrograph of AC with a small focus of comedo-type necrosis within the tumor nest. Magnification: 100X. **(B)** High magnification view of AC with a mitotic figure in the center of this photo. Tumor cells show neuroendocrine cytologic features, including a moderate amount of eosinophilic cytoplasm and finely granular nuclear chromatin. Magnification: 400X. H&E stain **(A, B)**.

AC, also known as intermediate-grade NETs or grade 2 NETs, is the most common type of tNENs. Around 40-50% of tNENs are AC. The clinical presentations, radiological manifestations, immunoprofile, and molecular alterations of AC are similar to those of TC (22). AC differs from TC only by microscopic features and clinical prognosis. AC is defined by increased mitotic counts (2-10 mitoses/2 mm^2^) and/or spot necrosis (**Figures 2A, B**). The Ki-67 index is elevated compared to TC, oftentimes up to 30%, although it is not useful to tell AC from TC AC is also characterized by a low mutational burden, but a slight overall increase from TC. Complete surgical resection or generous incisional/excisional biopsy specimen, extensive gross sampling, and careful microscopic examination are usually required to establish the diagnosis of AC. AC usually cannot be differentiated from TC on cytology specimens or small biopsies. In such instances, the diagnosis should be rendered as carcinoid tumor, with a note detailing the mitotic counts and presence or absence of necrosis in the limited samples, stating that the differential includes TC and AC, and recommending complete surgical resection for further evaluation.

The treatment options of AC are similar to those of TC. The rates of lymph node and distant metastasis are higher than those of TC. The 5-year survival of thymic AC is variable, ranging from 20% to 87.5%, with a median survival of 59 months (1, 19, 20, 22–26).

## Large cell neuroendocrine carcinoma

**Figure 3:**
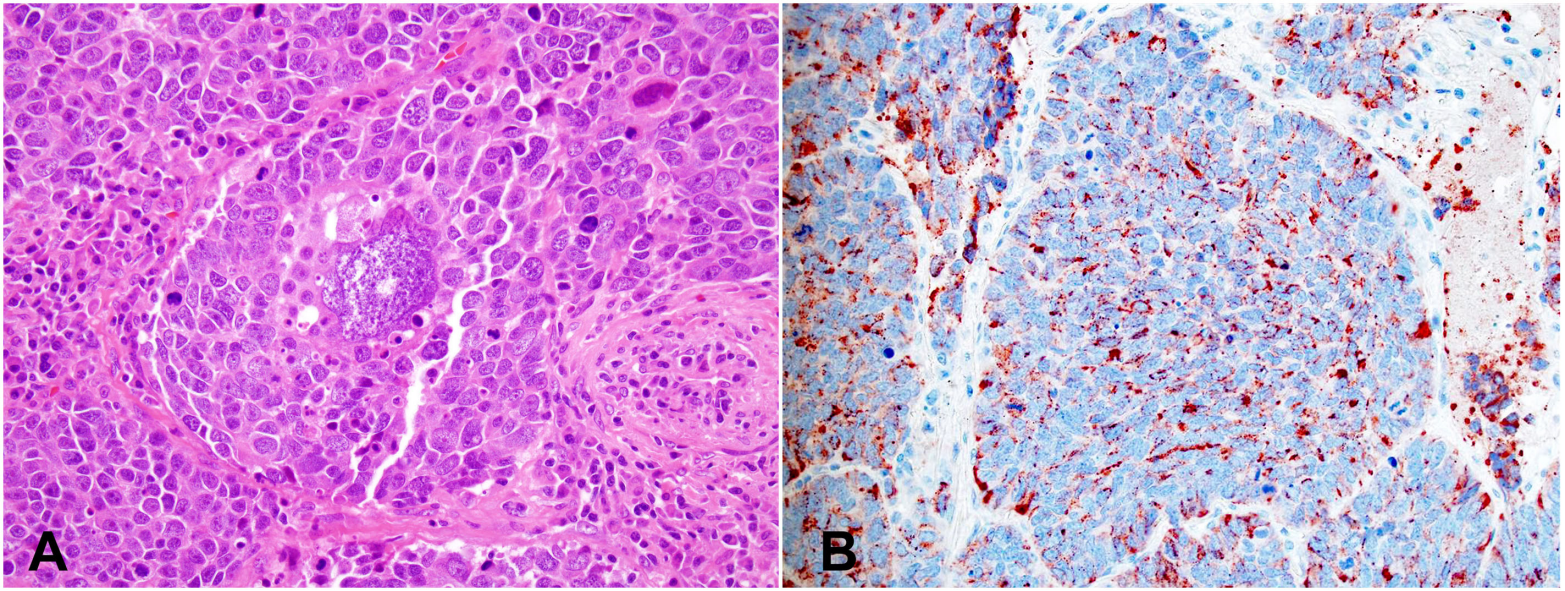
Large cell neuroendocrine carcinoma (LCNEC). **(A)** High magnification view of LCNEC comprising large pleomorphic tumor cells with a moderate amount of eosinophilic cytoplasm, coarse chromatin, and prominent nucleoli. Frequent mitoses and apoptotic bodies are seen. The tumor shows a nested growth pattern with focal peripheral palisading. H&E stain. **(B)** Immunohistochemical stain of Chromogranin A is diffusely positive in the tumor cells. Magnification **(A, B)**: 400X. *Photographs are courtesy of Jeffrey L. Myers, MD, University of Michigan, Ann Arbor, MI*.

Thymic LCNEC is more common in males, with a median age of 57 years. Ture thymic LCNEC is rare. The indistinct diagnostic criteria may have attributed to the higher incidence rates in some reports (27–29). LCNEC is a high-grade tumor with neuroendocrine morphological features and reactivities to at least one of the neuroendocrine immunohistochemical markers (**Figures 3A, B**). The neuroendocrine architectures of LCNEC are often less prominent or organized compared to those of TC and AC. The reactivities to immunohistochemical markers need to be strong and diffuse in cases of LCNEC with less prominent neuroendocrine architectures, since weak and nonspecific staining of some neuroendocrine markers are not uncommon in other tumors such as adenocarcinoma and thymic carcinoma with neuroendocrine differentiation. The tumor cells show nonsmall cell cytological features, including moderate to abundant cytoplasm and pleomorphic nuclei with coarse chromatin and prominent nucleoli. High mitotic count (>10, often >20 mitoses/2 mm^2^), frequent apoptosis, and extensive necrosis are commonly seen. The Ki-67 index is high (>30%, generally 40-80%).

Under the current WHO classification, a subset of LCNEC is described as AC-like tumors with increased mitotic counts above the threshold of AC (between 11 to 20 mitoses/2mm^2^). These tumors show similar although slightly higher mutation burdens and chromosomal alterations compared to AC and TC. Still, these molecular profiles are significantly different from those of the high-grade NET, including LCNEC and SmCC (4, 30). This subset of tumors is predicted to behave less aggressively than the other LCNEC, resembling the concept of grade 3 NET of the pancreas (31–33). However, there are insufficient data to define this grade 3 NET category in the thymus or the lungs. Ongoing and future studies may supply evidence to separate this subset from LCNEC in the future. The current recommendation for these tumors is to add a comment to describe the AC-like morphological features and mitotic counts, and to suggest less aggressive managements than ordinary LCNEC.

The main differential diagnosis of LCNEC is thymic carcinoma with neuroendocrine differentiation. The latter does not demonstrate any neuroendocrine architecture. Immunohistochemistry can also be helpful in this differential. The thymic carcinomas with neuroendocrine differentiation usually only show focal and weak neuroendocrine immunoreactivities, and they are commonly positive for CD117, CD5, and p63. In contrast, neuroendocrine immunoreactivities in LCNEC are generally more diffuse compared to those in thymic carcinoma, although focal expression of NE markers is sufficient for diagnosing LCNEC when the NE architecture is as prominent. LCNEC can be focally positive for CD117 but generally negative for CD5 and p63 (1, 34, 35).

The prognosis of LCNEC is worse than that of TC and AC. The 5-year survival rate varies from 0% to 66% in the literature (4, 20, 35, 36).

## Small cell carcinoma

SmCC is the uncommon but most aggressive form of tNENs, accounting for less than 10% of all tNENs (1, 20, 35, 37). Most patients with SmCC present with symptoms such as chest pain, weight loss, night sweating, and superior vena cava syndrome. Paraneoplastic manifestations such as Cushing syndrome and myasthenic syndrome are extremely rare (38, 39). Thymic SmCC is equally seen in males and females. The pathogenesis is unknown, and smoking has not been proved as a risk factor.

**Figure 4:**
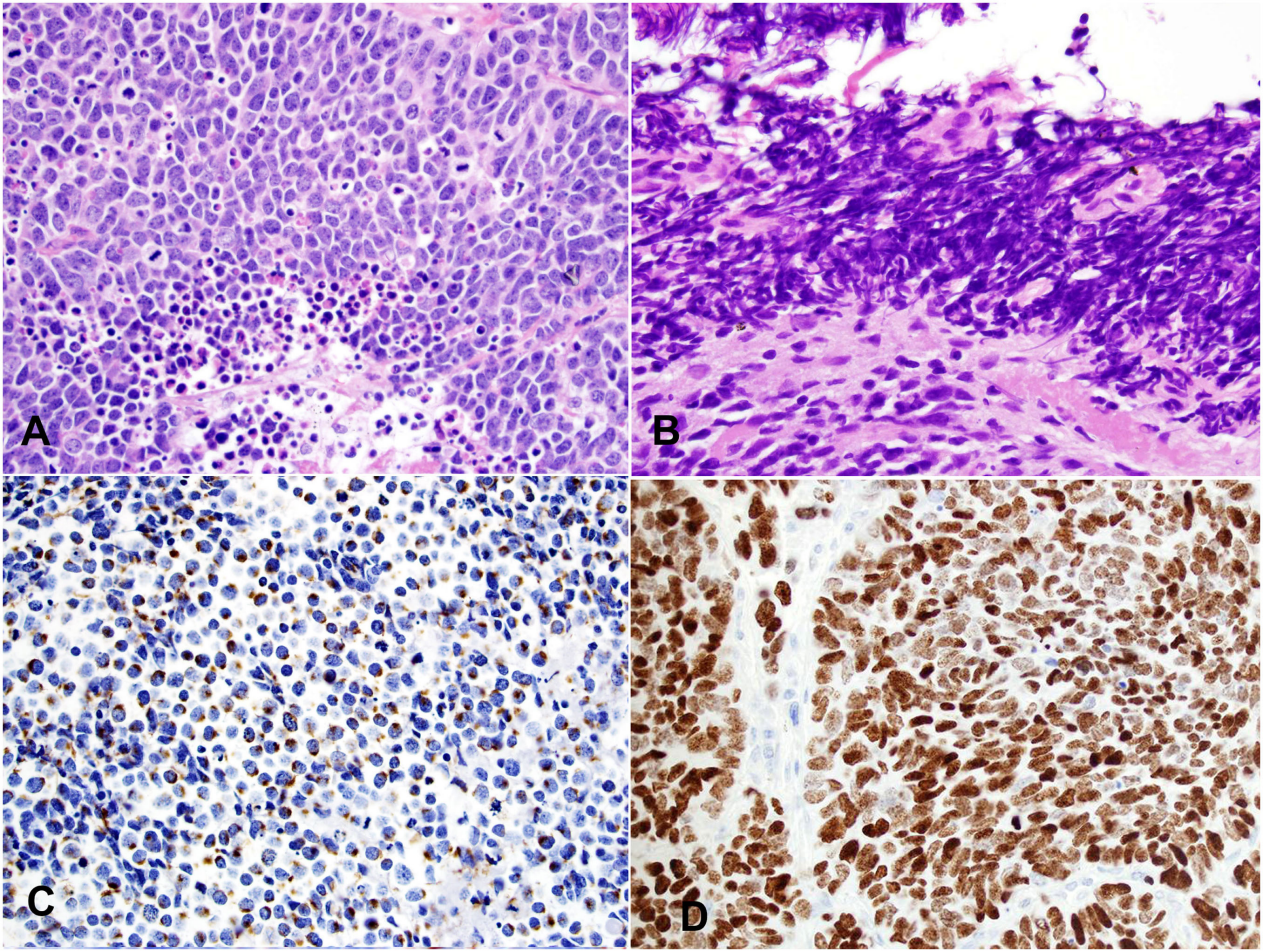
Small cell carcinoma (SmCC). **(A)** High magnification view of SmCC showing densely packed small to intermediate-sized tumor cells with scant cytoplasm, dense chromatin, and occasional inconspicuous nucleoli. Numerous mitoses and apoptotic bodies are seen. **(B)** One area of the tumor shows a prominent crushing artifact, a common feature of SmCC. H&E stain **(A, B)**. **(C)** The tumor cells show characteristic cytoplasmic perinuclear dot-like staining for cytokeratin Cam 5.2. **(D)** The Ki-67 index is high (almost 100%). Magnification **(A-D)**: 400X.

Macroscopically, SmCC is usually large in size (most >10 cm) with invasive growth. Tumor frequently invades into adjacent structures such as lung, pleura, pericardium, and large vessels. The microscopic features are like SmCC of all other organs. The size of the tumor cell is about three times the size of a resting lymphocyte. Tumor cells show a high nuclear-to-cytoplasmic ratio with nuclear molding and crushing artifacts (**Figures 4A, B**). The nuclei are oval with fine, granular chromatin and small, inconspicuous nucleoli. Mitotic counts are high (usually >50 mitoses/2mm^2^). Numerous apoptotic bodies and extensive necrosis are standard features of SmCC. Most cases of thymic SmCC stain positively for cytokeratins, typically in a peri-nuclear dot-like pattern (**Figure 4C**). Patchy reactivities to neuroendocrine markers such as synaptophysin and chromogranin A are common but are not required for the diagnosis. The Ki-67 index is high (commonly 80-100%, **Figure 4D**).

The main differential diagnosis of thymic SmCC is metastasis or direct invasion from pulmonary SmCC. Clinical information and radiographic findings are the keys to this differential. TTF-1 immunohistochemical stain is not helpful in this differential since thymic SmCC, just like SmCC from other extrapulmonary organs, can be positive for TTF-1 (40). Other differential diagnoses include mediastinal T lymphoblastic leukemia/lymphoma (TLL) and sarcomas such as Ewing’s sarcoma and rhabdomyosarcoma. Immunohistochemistry studies and fluorescence *in situ* hybridization (FISH) tests are helpful to exclude those differentials, e.g., CD45, TDT, and CD3 for TLL, CD99, TLE1, and EWSR1 translocation for Ewing’s sarcoma, and myogenin and myoD1 for rhabdomyosarcoma. SmCC invariably demonstrates high mutational burdens characterized by severe gene alterations such as biallelic inactivation of *RB1* and *TP53*.

Most patients with thymic SmCC present with advanced clinical stages and are inoperable. The overall response to chemoradiotherapy is limited. The prognosis of thymic SmCC is poor. The reported 5-year survival rate is 0%, with a median survival of only 14 months (20, 35, 41–43).

## Conclusion and future perspectives

All categories of tNENs have been described in other organs, although tNENs show some subtle distinctions such as the underlying risk factors (relationship to smoking) and relative frequency of each tumor type. Limited available data on tNENs have demonstrated similar clinical behavior to their respective counterparts, such as pulmonary and pancreatic NETs. It is reasonable to predict that future updates on the classification and clinical management of tNENs will follow the leads of NETs of other organs. However, many open questions on the biologic aspects of tNENs remain to be studied to better understand and treat these rare tumors.
